# Pyrazinamide resistance and mutations in *pncA* among isolates of *Mycobacterium tuberculosis* from Khyber Pakhtunkhwa, Pakistan

**DOI:** 10.1186/s12879-019-3764-2

**Published:** 2019-02-06

**Authors:** Muhammad Tahir khan, Shaukat Iqbal Malik, Sajid Ali, Nayyer Masood, Tariq Nadeem, Anwar Sheed Khan, Muhammad Tanvir Afzal

**Affiliations:** 1 Department of Bioinformatics and Biosciences, Capital University of Science and Technology, Islamabad Expressway, Kahuta Road, Zone-V, Islamabad, Pakistan; 2 Department of Microbiology, Quaid-E-Azam University Islamabad and Provincial Tuberculosis Reference, Laboratory Hayatabad Medical Complex, Peshawar, Pakistan; 3 Department of Computer Science, Capital University of Science and Technology, Islamabad, Pakistan; 4 National Center of Excellence in Molecular Biology, University of The Punjab, Lahore, Pakistan; 5 Institute of Basic Medical Sciences, Khyber Medical University and Provincial Tuberculosis Control Laboratory Hayatabad Medical Complex Peshawar, Peshawar, Pakistan

**Keywords:** Pyrazinamide, Resistance, Mutations, *pncA*

## Abstract

**Background:**

Pyrazinamide (PZA) is an important component of first-line drugs because of its distinctive capability to kill subpopulations of persistent *Mycobacterium tuberculosis* (MTB). The prodrug (PZA) is converted to its active form, pyrazinoic acid (POA) by MTB *pncA*-encoded pyrazinamidase (PZase). Mutation in *pncA* is the most common and primary cause of PZA resistance. The aim of the present study was to explore the molecular characterization of PZA resistance in a Pashtun-dominated region of Khyber Pakhtunkhwa, Pakistan.

**Methods:**

We performed drug susceptibility testing (DST) on 753 culture-positive isolates collected from the Provincial Tuberculosis Control Program Khyber Pakhtunkhwa using the BACTEC MGIT 960 PZA method. In addition, the *pncA* gene was sequenced in PZA-resistant isolates, and PZA susceptibility testing results were used to determine the sensitivity and specificity of *pncA* gene mutations.

**Results:**

A total of 69 isolates were PZA resistant (14.8%). Mutations were investigated in 69 resistant, 26 susceptible and one H37Rv isolates by sequencing. Thirty-six different mutations were identified in PZA-resistant isolates, with fifteen mutations, including 194_203delCCTCGTCGTG and 317_318delTC, that have not been reported in TBDRM and GMTV Databases and previous studies. Mutations Lys96Thr and Ser179Gly were found in the maximum number of isolates (*n* = 4 each). We did not detect mutations in sensitive isolates, except for the synonymous mutation 195C > T (Ser65Ser). The sensitivity and specificity of the *pncA* sequencing method were 79.31% (95% CI, 69.29 to 87.25%) and 86.67% (95% CI, 69.28 to 96.24%).

**Conclusion:**

Mutations in the *pncA* gene in circulating isolates of geographically distinct regions, especially in high-burden countries, should be investigated for better control and management of drug-resistant TB. Molecular methods for the investigation of PZA resistance are better than DST.

## Background

Tuberculosis (TB) is a common life-threatening infectious disease caused by *Mycobacterium tuberculosis* (MTB) [1]. The standard new therapy includes a six-month treatment of four recommended first-line drugs, i.e., isoniazid, rifampin, pyrazinamide and ethambutol [2]. However, the misuse of these antibiotics has led to the emergence of multidrug-resistant (MDR) strains of MTB [3]. According to the WHO report in 2016, Pakistan is ranked among the top five countries, with a prevalence of 56% of global TB and among the high-burden drug-resistant countries [4]. The incidence of drug resistance (MDR/RR-TB) is twenty-six thousand (16–36) with 14 (8.5–19) per hundred thousand individuals in a population. This situation leaves a high challenge to the TB control program in the country.

Khyber Pakhtunkhwa (KPK), a region in Pashtun, is the third largest province of Pakistan, with an area of 74,521 km^2^ and a population of approximately 30,523,371 individuals. Tuberculosis remains a major public health problem and one of the neglected health areas in the past. Recently, a TB control program has been launched at Hayatabad Medical Complex Peshawar that regularly monitors the incidence of TB as well as drug susceptibility testing (DST) in the population.

Pyrazinamide (PZA), a key first-line antibiotic used for the short-course treatment of 6 months, kills dormant tubercle bacilli at an acidic pH, whereas other drugs fail to work during the early severe stages of chemotherapy [5]. Due to some technical and buffering issues of drug susceptibility testing (DST) for PZA, the results of phenotypic resistance are not always reliable [6]. The MGIT 960 system is the most reliable method to perform PZA-DST [7, 8] and is currently the only available phenotypic test method to explore PZA susceptibility. MTB isolates are cultured in the presence of PZA under acidic conditions as required for conversion into pyrazinoic acid (POA) [8], the active form of pyrazinamide in vivo, activated by MTB PZase (*PncA*). These issues have prompted efforts towards molecular methods of PZA resistance [9].

Mutations occurring in the *pncA* gene are most commonly associated with pyrazinamide (PZA) resistance [9–12].

In Khyber Pakhtunkhwa, there are no molecular studies to explore *pncA* mutations in the KPK Pashtun-dominant region. Here, we aimed to compare phenotypic resistance to PZA to genotype and identify mutations in *pncA* among PZA-resistant isolates circulating in this epidemiologically distinct area in a Pashtun-dominant population, which may be useful in tracing the transmission in patients.

## Material and methods

### Ethical considerations

The present investigation was approved by the Institutional Ethics Committee of CUST Islamabad and Provincial Tuberculosis Reference Laboratory (PTRL) KPK under reference number PTP/PTRL-402/16. Prior to the study, informed consent was obtained from each TB patient, however, the results were not linked back to individual patients.

### Study samples

All samples were processed at the BSL-III facility of PTRL, Hayatabad Medical Complex (HMC). The lab receives TB cases from the entire province, which is facilitated by the MGIT 960 system of drug susceptibility testing. The data for TB patients were collected from their guardians or caretakers.

### Sample processing, isolation and mycobacterial culture

The samples were processed using the N-acetyl-L-cysteine–sodium hydroxide (NALC–NaOH) concentration method [13] in a Falcon tube containing an equal volume of the NaOH/N-acetyl-L-cysteine (NALC), which was subsequently vortexed and incubated at room temperature for 15 min for decontamination and digestion. Next, 50 ml of phosphate buffer was transferred to each tube, followed by centrifugation at 3000 rpm for 15 min. The supernatant was transferred to a fresh tube containing 5% phenol, while the pellet was mixed with phosphate buffer and cultured on Lowenstein–Jensen medium (LJ) in MGIT tubes containing 7H9 media.

### Drug susceptibility testing (DST)

Drug susceptibility testing of PZA was performed through the automated BACTEC MGIT 960 system (BD Diagnostic Systems, NJ, USA) [14]. *Mycobacterium tuberculosis* H37Rv and *Mycobacterium bovis* were used as susceptible and resistant controls, respectively. A sample was marked as PZA resistant if growth was found at 100 μg/ml of the PZA critical concentration. DST for resistant isolates was repeated for confirmation of drug resistance. The PZA-resistant samples were further subjected to DST with isoniazid (INH), rifampin (RIF), ethambutol (EMB), amikacin (AMK), streptomycin (SM), capreomycin (CAP), ofloxacin (OFX) and kanamycin (KM) through the BACTEC MGIT 960 system, with critical concentrations of drugs as per the policy guidelines of the WHO (WHO 2014) [15]. The resistant samples were further manually assessed to confirm the growth of MTB against the critical drug concentration.

### DNA extraction, PCR amplification and sequencing

Genomic DNA from PZA-resistant isolates were extracted by sonication [16, 17]. One microliter of fresh culture was transferred from a Mycobacterium Growth Indicator Tube (MGIT) to a microcentrifuge tube and boiled at 86 °C for 30 min using an Echotherm™ IC22 Digital, Chilling/Heating Dry Bath followed by 15 min sonication using a sonicator (ELMASONIC S30). All the samples were centrifuged for 5 min at 10,000 rpm. The supernatant containing DNA was stored at − 20 °C. The fragments containing *pncA* were amplified using previously reported primers (*pncA*-F = 5GCGTCATGGACCCTATATC-3 and *pncA*-R = 5 AACAGTTCATCCCGGTTC-3=) [18]. Each 50-μl PCR contained 0.1 μl of each DNTs, 0.8 μl of Taq (New England Biolabs, UK)), 5 μl of PCR buffer, 3 μl of MgCl_2,_ 1 μl of each forward and reverse primer, 34.8 μl of molecular grade water and 4 μl of genomic DNA. The PCR conditions were 5 min at 94 °C for denaturation, followed by 30 cycles of 30 s at 94 °C, 30 s at 56 °C, and 72 °C for 1 min, with an extension step at 72 °C for 5 min, as previously described. The PCR product was analyzed by 6 Applied Biosystems 3730xl (Macrogen Korea)**.**

### Data analysis

The sequence data obtained were loaded into Mutation Surveyor V5.0.1 software [19]. The data were analyzed and compared with the *PncA* (Rv2043c) gene of RefSeq database of NCBI (NC_000962), while the patient data were entered by using Epi-Data entry version 3.1 software and analyzed through Epi-Data analysis software.

## Results

### Socio-demographic characteristics

A total of 4518 samples were collected from TB subjects from all districts of KPK. Among these individuals, 753 subjects were culture positive, with ages ranging from 8 to 76 years (median age = 34.34). A majority of the cases were never treated (diagnostics) (44/69). All patients were KPK residents with Pushto as the main language (Table 1).

**Table 1:** Drug susceptibility profile and socio-demographic data of 69 PZA resistant patients

Resistant Type	Number								
MDR	52								
Mono_Resistant	1								
Poly_Resistant	10								
XDR	6								
History	Resistants								
Diagnostic	47								
Follow Up	22								
Disease Type	Resistants								
Extra Pulmonary	3								
Pulmonary	66								
Sample Type									
	BAL	Pus	Sputum						
Resistant	1	2	66						
Gender	Resistant								
Female	44								
Male	25								
Resistant level of other drugs in PZA resistance isolates									
	INH	RIF	EMB	OFX	SM	KAN	CAP	AM	MOX
Resistant	64	58	35	35	28	13	11	11	2
sensitive	5	11	34	34	40	56	58	58	67

*AMK* Amikacin, *BAL* Bronchoalveolar Lavage, *CAP* Capreomycin, *EMB* Ethambutol, *INH* Isoniazid, *KM* Kanamycin, *MDR* multidrug resistance, *OFX* Ofloxacin, *PZA* pyrazinamide, *RIF* rifampin, *SM* streptomycin, *XDR* extensively drug resistance

### Drug susceptibility pattern

By using the BACTEC MGIT 960 system, 69 (14.8%) isolates were classified as PZA resistant. All PZA resistant isolates, 26 PZA-sensitive isolates and one H37Rv isolate were sequenced to analyze the mutations in the 561 bp region of *pncA*. Multidrug drug resistance (MDR) and extreme drug resistance (XDR) were detected in 52/69 (75.35%) and 6/69 (8.69%) isolates, respectively.

Risk factors such as age, gender, history, reason, disease type and resistance type are presented in Table 1. The presence of a high number of MDR isolates (52/69) in PZA-resistant isolates shows the major risk factor in transmission and treatment failure.

### Mutation in PZA-resistant and PZA-susceptible isolates

Mutations were investigated in both resistance and susceptible isolates in the coding region (561 bp) of *pncA* (Table 2). Among the 69 PZA-resistant isolates 51 (74%), thirty-six different mutations with fifteen novel mutations, including 194_203delCCTCGTCGTG and 317_318delTC, were detected; but these variations were not found in TBDRM and GMTV Databases [20, 21]. The most common mutations detected at positions 287 and 423 were Lys96Thr (*n* = 4) and Ser179Gly (n = 4), respectively. A majority of these variations were substitution mutations, except for three deletions, 194_203 del CCTCGTCGTG (*n* = 1), 317_318delTC (n = 1), 530 del C (*n* = 3). A common synonymous mutation at position 195 C > T (Ser65Ser) was observed in both resistant (*n* = 22) and susceptible isolates (*n* = 16).

**Table 2:** Mutations in *pncA* gene of *M. tuberculosis* in 51 isolates

Nucleotide position	Codon Change	A.A change	Mutation Type	TBDReaMDB	GMTV Database	Reported (literature)	No. of isolates
33C > A	11.AAC / AAA	^u^ASN11LYS	Non-synonymous	Unreported	Unreported	No	1
35A > C	12.GAC/GCC	^a^ASP12ALA	Non-synonymous	reported	reported	–	2
53C > A	18.TCG/TAG	SER 18 STOP	non-sense	Unreported	Unreported	No	1
56 T > G	19.CTG/CGG	^u^LEU19ARG	Non-synonymous	reported	Unreported	–	1
137C > T	46.GCA/GTA	^u^ALA46VAL	Non-synonymous	reported	reported	–	1
161C > T	54.CCG/CTG	^a^PRO54LEU	Non-synonymous	reported	reported	–	1
170A > C	57.CAC/CCC	^a^HIS57PRO	Non-synonymous	reported	Unreported	–	1
194_203	Del CCTCGTCGTG	FRAMSHIPT	FRAMSHIPT	Unreported	Unreported	No	1
202 T > C	68.TGG/CGG	^a^TRP68ARG	Non-synonymous	reported	reported	–	1
205C > A	69.CCA/ACA	^u^PRO69THR	Non-synonymous	Unreported	Unreported	No	3
211C > T	71.CAT/TAT	^a^HIS71TYR	Non-synonymous	reported	reported	–	3
212A > G	71.CAT/ CGT	^a^HIS71ARG	Non-synonymous	reported	reported	–	1
226A > C	76.ACT/CCT	^a^THR76PRO	Non-synonymous	reported	reported	–	1
286A > C	96.AAG/CAG	^a^LYS96GLN	Non-synonymous	reported	Unreported	–	4
317–18	Del TC	^u^FRAMSHIPT	FRAMSHIPT	Unreported	Unreported	No	3
331G > T	111. GAG/TAG	STOP	Non-sense	Unreported	Unreported	No	1
359 T > G	120. CTG/CGG	^u^LEU120ARG	Non-synonymous	Unreported	reported	–	1
368G > C	123. CGC/CCC	^a^ARG123PRO	Non-synonymous	Unreported	reported	–	1
376G > A	126. GAT/AAT	^u^ASP126ASN	Non-synonymous	Unreported	Unreported	No	2
385G > A	129. GAT/AAT	^u^ASP129ASN	Non-synonymous	Unreported	Unreported	[7]	1
391G > T	131. GTC/TTC	^u^VAL131PHE	Non-synonymous	Unreported	reported	–	1
398 T > C	133. ATT/ACT	^a^ILEU133THR	Non-synonymous	Unreported	reported	–	2
419G > A	140. CGC/CAC	^u^ARG140HIS	Non-synonymous	Unreported	Unreported	No	2
422A > C	141. CAG/CCG	^u^GLN141PRO	Non-synonymous	Unreported	reported	–	3
430G > A	144. GAG/AAG	^u^GLU144LYS	Non-synonymous	Unreported	Unreported	No	1
437C > T	146. GCG/GTG	^a^ALA146VAL	Non-synonymous	reported	reported	–	1
449G > C	150. GGC/GCC	^u^GLY150ALA	Non-synonymous	Unreported	Unreported	No	1
461G > C	154. AGG/ACG	^u^ARG154THR	Non-synonymous	Unreported	Unreported	[36]	1
470 T > G	157. GTG/GGG	^a^VAL157GLY	Non-synonymous	Unreported	Unreported	[9, 37, 38]	2
508G > C	170. GCC/CCC	^u^ALA170PRO	Non-synonymous	Unreported	Unreported	No	1
519G > A	173. GAG/GAA	^u^GLU173GLU	synonymous	Unreported	Unreported	No	1
522G > A	174. GAG/GAA	^u^GLU173GLU	synonymous	Unreported	Unreported	No	1
530	DEL C	^u^FRAMSHIPT	FRAMSHIPT	Unreported	Unreported	No	3
535A > T	179. AGC/TGC	^u^SER179CYS	Non-synonymous	Unreported	Unreported	[39]	4
535A > G	179. AGC/GGC	^u^SER179GLY	Non-synonymous	Unreported	Unreported	No	1
538G > T	180. GTC/TTC	^u^VAL180PHE	Non-synonymous	reported	Unreported	–	1

*TBDReaMDB* Tuberculosis Drug Resistance Mutation Database

GMTV, Genome-wide *Mycobacterium tuberculosis* variation (GMTV): a, Mutations conferring PZA^r^ with very high confidence (category-A). u, mutations with unclear role (category D) (Miotto et al., 2014)

### Comparing phenotypic PZA-resistant with DNA sequencing

Among sensitive isolates we did not detect any nonsynonymous mutations in the coding region of *pncA*. To estimate the performance of DST compared with the *pncA* sequencing result, the genotypic data and phenotypic data for all 69 resistance isolates were evaluated. Considering phenotype as a reference, among the 69 resistant isolates, 51 (74%) isolates showed mutations, with sensitivity of 79.31% (95% CI, 69.29 to 87.25%) and specificity of 86.67% (95% CI, 69.28 to 96.24%).

## Discussion

PZA is a distinctive antituberculosis drug that plays a key role in shortening TB treatment. PZA kills nonreplicating persistent MTB and is prescribed in both susceptible and MDR-TB treatment. After conversion into its active form POA by pyrazinamidase (PZase), PZA remains active at low pH during acidic stress [11]. However, in a large number of cases, MTB patients develop resistance against PZA that led to the survival of persistent bacteria. Conventional methods of PZA DST increase the level of false resistance that may result from media buffering issues and large inoculum sizes, where the acidic environment is required for drug action but inhibits the growth of MTB [6, 22, 23]. Under such conditions, the most reliable method in the present scenario is the molecular detection of PZA-resistance, which involves sequencing the *pncA* gene to assess mutations in the 561 bp coding region and upstream regulatory region. In the present study, more than half of the tested PZA-resistant isolates were also MDR-TB, 52/69 (75.35%) isolates, consistent with the results of previous studies [12, 24]. Previous studies [25, 26] have also shown a correlation between mutations in the *pncA* gene and phenotypic PZA-resistance. Based on these findings, we report mutations in 51 (74%) resistant isolates that harbor 36 mutations in the coding region of *pncA*, with sensitivity and specificity of *pncA* sequencing of 79.31% (95% CI, 69.29 to 87.25%) and 86.67% (95% CI, 69.28 to 96.24%), respectively. Streicher et al. and Whitfield reported better sensitivity and specificity for *pncA* sequencing compared with that of MGIT 960 DST of 90.9, 100% [27] and 95.0 (95% CI 92.1–98.0), 99.1 (95% CI 98.4–99.9) [28]. Miotto et al. (2014) identified 280 mutations in 1950 clinical strains [9], which were categorized into four groups, 1) very high confidence resistance mutations, 2) high-confidence resistance mutations, 3) mutations with an unclear role and 4) mutations not associated with phenotypic resistance based on confidence level. We detected 12 mutations with very high confidence resistance, while the rest of mutations detected have been found in Miotto unclear category (Table 2). The mutations 211C > T, 212A > G, 226A > C, 286A > C and 422A > C in the present study (Table 2) were previously shown as very high confidence resistance mutations [9, 12, 29]. Molecular biomarkers that could specifically target the first two categories should be developed [9].

Tan et al. (2014) reported that each geographical region has a diverse type of variations in *pncA*. Isolates from Southern China exhibited a scattered type of mutations in 561 bp region, which remains a complex target in the development of diagnostic biomarkers in identification of all resistance conferring mutations [26]. Some strains, which were PZA resistant by conventional DST, lack any mutations in *PncA* and its regulatory gene, suggesting other targets of drug and issues concerning DST.

The residues Cys138, Asp8, Lys96 and Asp49, His51, His57, and His71 are present in the active and metal binding sites [30, 31] of the *pncA*-encoded enzyme pyrazinamidase (PZase). We identified mutations dispersed throughout the *pncA* gene (35A > C---538G > T) nearby the area of metal binding and active site amino acids (46–76 and 133–146). We detected mutations that are important for enzyme catalysis and metal binding (Table 2). However, we did not detect any mutations in the 18 PZA-resistant MTB isolates, suggesting the involvement of other genes RpsA and PanD (aspartate decarboxylase) [32, 33]. A potential new target of PZA, the clpC1 (Unfoldase) gene, which encodes a family of ATPases, was identified in PZA-resistance isolates in addition to the previously identified genes *pncA*, rpsA and panD [34]. However, the role of these genes (rpsA, panD and clpC1) in PZA resistance is small compared to that of *PncA*. In a more recent study four new efflux proteins Rv0191, Rv1667c, Rv3756c and Rv3008 were implicated in PZA/POA resistance [35]. These findings suggest a new mechanism for PZA resistance in MTB. Further investigations are needed to identify the quantitative role of all these targets and mechanisms in PZA-resistant MTB for better management of drug-resistant TB.

In conclusion, considering phenotype as a reference, among the 69 PZA resistant isolates, 51 (74%) showed mutations with sensitivity of 79.31% (95% CI, 69.29 to 87.25%) and specificity of 86.67% (95% CI, 69.28 to 96.24%). The mutations 33C > A, 53C > A, 194_203 Del CCTCGTCGTG, 205C > A, 317–18 Del TC, 331G > T, 376G > A, 419G > A, 430G > A, 449G > C, 508G > C, 519G > A, 522G > A, 530DEL C and 535A > G were not found in the GMTV and TBDRM databases and neither in previous studies suggesting the need of some more studies from distinct geographical regions must be carried out for some novel mutations confined to that specific areas. Majority of mutations were of high confidence intervals and uncharacterized category in resistance. Molecular methods to investigate PZA resistance by screening mutations in *pncA* gene in distinct epidemiological regions offer a much more rapid alternative compared to that of conventional bacteriology. Mutations in *pncA* gene are highly linked with resistance to PZA and scattered in the entire coding region of *pncA*. Further, we found an association between PZA resistance and resistance to other important first line drugs, INH and RIF, which is a major hurdle in the treatment management of MDR TB. This high frequency of *pncA* mutations from geographically distinct regions recommends the WHO guidelines to empirically use the pyrazinamide in drug-resistant TB should be considered. Further studies with large sample size may strengthen these findings to identify the mutations in PZA-resistant isolates specific to certain geographical areas for the better treatment and development of geographically specific biomarkers.

## Abbreviations

AMK: Amikacin
CAP: Capromycin
CIs: Confidence intervals
DST: Drug susceptibility testing
EMB: Ethambutol
GMTV: Genome-wide *Mycobacterium tuberculosis* variation
INH: Isoniazid
KAN: Kanamycin
KPK: Khyber Pakhtunkhwa
MDRTB: Multidrug-resistant tuberculosis
OFLX: Ofloxacin
POA: Pyrazinoic acid
PTRL: Provincial tuberculosis control program
PZA: Pyrazinamide
PZase: Pyrazinamidase
RIF: Rifampicin
SM: Streptomycin
TBDReaMDB: Tuberculosis Drug Resistance Mutation Database
XDR: Extreme drug resistance
